# Gut and Orbital Dysbiosis Associated with Graves’ Disease and Graves’ Orbitopathy: A Systematic Review

**DOI:** 10.3390/jcm15124586

**Published:** 2026-06-12

**Authors:** Abdel Mohaimen Missaoui, Oumeyma Trimeche, Ekram Hajji, Helena Mosbah

**Affiliations:** 1 Department of Endocrinology-Diabetology-Nutrition, Poitiers University Hospital, 86000 Poitiers, France; 2 University of Poitiers, 86000 Poitiers, France; 3 Department of Endocrinology-Diabetology-Nutrition, Angoulême Hospital Center, 16000 Angoulême, France; 4 Department of Internal Medicine and Endocrinology, Monastir University Hospital, Monastir 5000, Tunisia

**Keywords:** Graves’ disease, Graves’ orbitopathy, autoimmune thyroid disease, gut microbiota, orbital microbiota, systematic review

## Abstract

**Background/Objectives:** Graves’ disease (GD) is a prevalent autoimmune thyroid disorder marked by thyrotoxicosis, goiter, and Graves’ orbitopathy (GO). Recent studies highlighted its association with dysbiosis. This systematic review aims to update the current literature and clarify the distinctive microbial signatures and dysbiosis associated with GD/GO. **Methods**: A systematic search for relevant studies was conducted across multiple databases (2000–2023), employing appropriate keywords. Relevant data were extracted from 25 eligible studies. **Results**: Microbiota analysis from 19 GD studies (713 patients, 546 controls) and eight GO studies (356 patients, 187 controls), primarily conducted in China (21/25), were examined. The gut microbiota richness and evenness were reduced in GD patients compared to controls in 62.5% of fecal samples. No consistent pattern in alpha diversity was observed in GO. Significant taxonomic divergence was observed between GD/GO and controls. At the phylum level, the Firmicutes to Bacteroidetes ratio was consistently decreased in GD patients (66.7%). Most GO patients also exhibited a similar disequilibrium in their gut and orbital adipose microflora. At the genus level, *Prevotella* (11 studies), genera within the Lactobacillaceae family (three studies), and *Streptococcus* (three studies) consistently showed an increase. Genera from the families Lachnospiraceae (nine studies), Ruminococcaceae (six studies), and Veillonellaceae (five studies), as well as the genus *Bacteroides* (three studies), were decreased. **Conclusions**: GD/GO-associated dysbiosis is characterized by reduced microbial richness and evenness and alterations in gut phyla balance (↓ Firmicutes, ↑ Bacteroidetes, ↑ Proteobacteria). Specific genera—including *Lactobacillus*, *Prevotella*, *Bacteroides*, and members of the Lachnospiraceae family—may plausibly act as contributors to the onset or progression of GD/GO by influencing the Th17/Treg balance, although their exact roles remain uncertain and largely hypothetical. **Systematic review registration: PROSPERO identifier CRD42024512007.**

## 1. Introduction

Graves’ disease (GD) is the primary cause of hyperthyroidism, affecting about 20 cases per 100,000 individuals annually. GD presents as an organ-specific autoimmune disorder characterized by thyrotoxicosis, goiter, and exophthalmos [1,2,3]. The root cause of GD lies in the breakdown of immune tolerance towards the thyroid-stimulating hormone receptor (TSHR), leading to the abnormal production of stimulating thyroid receptor autoantibodies (TRAb). These TRAb bind to TSHR and mimic the action of this hormone, triggering downstream effects such as thyrocyte proliferation, thyroid enlargement, and increased secretion of thyroid hormones [1,3,4]. Graves’ orbitopathy (GO), also known as thyroid eye disease, is the major extrathyroidal manifestation of GD. Approximately 25–50% of GD patients experience GO, with an estimated prevalence of around 9 per 10,000 individuals in the general population [5]. The onset of GO is precipitated by the combined action of TRAb and insulin-like growth factor 1 receptor, leading to the differentiation of orbital preadipocytes into adipocytes. This process fosters the expansion of orbital adipose tissue, which underlies the clinical manifestations of the disease [4,5,6].

This autoimmune dysregulation is a complex interplay influenced by various factors, including genetics, endogenous, and environmental factors. Traditional environmental elements such as viral infections, smoking, stress, and iodine intake are recognized for their substantial roles in initiating or worsening GD and GO [2,3]. Nonetheless, recent studies have highlighted the emerging influence of the human microbiome in the onset and progression of autoimmune thyroid disorders (AITDs) [7,8,9]. Similar findings have been observed in a range of conditions, including cardiometabolic disorders, neurodegenerative diseases, and autoimmune conditions such as type 1 diabetes mellitus, rheumatoid arthritis, multiple sclerosis, and inflammatory bowel disease (IBD) [10,11,12,13].

In humans, the gut harbors the largest quantities of microorganisms and the greatest number of species compared to other parts of the body (see Appendix A) [14,15]. Key roles of the gut microbiota include metabolizing dietary elements into bioactive food components and producing short-chain fatty acids (SCFAs) such as acetic, propionic, and butyric acids. Additionally, they play a crucial role in protecting intestinal surfaces, thereby maintaining stability and preventing the invasion of pathogenic microorganisms. The influence of gut microbiota on the host’s immune system is substantial, contributing significantly to the maturation of both adaptive and innate immunity [12,13,14,15].

While previous systematic reviews have underscored the profound association between disruptions in human microbiota balance, referred to as dysbiosis, and AITDs, there remains a paucity of literature focusing specifically on the microbial alterations observed in GD/GO [16]. Our systematic review aims to update the current literature and clarify the distinctive microbial signatures and dysbiosis associated with GD/GO.

## 2. Methods

This systematic review adheres to the Preferred Reporting Items for Systematic Reviews and Meta-Analyses (PRISMA) guidelines, with its protocol registered in PROSPERO [CRD42024512007]. The PRISMA 2020 checklist is provided in the Supplementary Materials (Table S1).

### 2.1. Search Strategy and Data Extraction

A comprehensive literature search for relevant studies published in English was conducted across multiple databases, including PubMed, Embase, Web of Science, and Google Scholar, spanning the period from January 2000 to December 2023. The search strategy employed the following combination of keywords: (“Graves’ disease” OR “Graves’ hyperthyroidism” OR “Graves’ orbitopathy” OR “Graves’ ophthalmopathy” OR “thyroid eye disease”) AND (“microbiota” OR “microbiome”).

The study selection and screening workflow was conducted using the Covidence (Veritas Health Innovation, Melbourne, Australia) online systematic review platform. After creating the review project and defining the review type, all search results retrieved from PubMed, Embase, Google Scholar, and Web of Science were imported into Covidence in RIS or CSV format. The platform automatically identified and removed duplicate records prior to screening. Title and abstract screening was performed independently by two authors, AM and OT, according to predefined inclusion and exclusion criteria, with Covidence flagging any conflicts for subsequent resolution. Full-text screening followed, during which PDFs were uploaded, and eligibility decisions were recorded with justifications.

Disagreements between authors (AM and OT) at both the title/abstract screening and full-text review stages were managed using the conflict resolution workflow within Covidence. All records were screened independently by two reviewers. Discrepancies were automatically identified by the platform and resolved through discussion and consensus, with arbitration by a third author (EH) when necessary. No statistical measures such as Cohen’s kappa were calculated, as the predefined dual-independent screening and structured conflict resolution process were considered sufficient to ensure methodological rigor and consistency in study selection.

**Figure 1:**
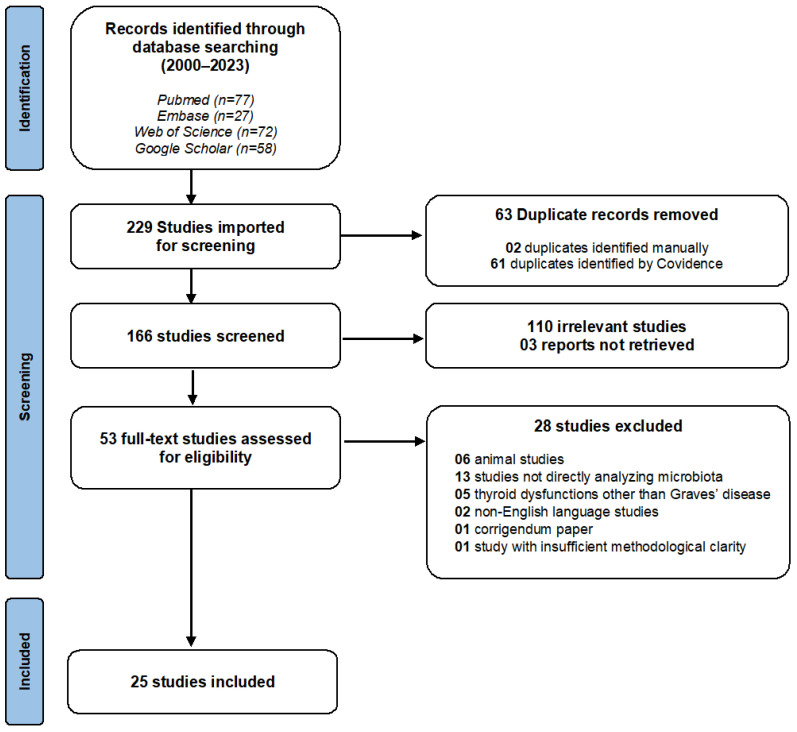
PRISMA-based illustration of our systematic literature review.

A meta-analysis was not conducted because the included studies exhibited substantial methodological heterogeneity, particularly in sequencing platforms, diversity metrics, and taxonomic reporting. These inconsistencies precluded any meaningful quantitative synthesis. The search process is depicted in Figure 1.

### 2.2. Inclusion and Exclusion Criteria

This study followed the PICOS parameters summarized in Table S2:

▪ Population: We included adult patients diagnosed with GD according to conventional criteria and/or GO according to EUGOGO criteria [5]. We excluded animal studies, individuals under 18 years old, pregnant or breastfeeding women, and those with other thyroid diseases or orbital conditions. Exclusions also comprised individuals with conditions influencing microbiota, severe diseases, extreme diets, or substance abuse.
▪ Intervention: Our study included newly diagnosed, untreated patients as well as those treated with antithyroid drugs (ATDs). We excluded patients taking medications that could affect the microbiota.
▪ Comparison: We considered healthy controls (HCs) or patients with varying severity grades of GD/GO for comparison.
▪ Outcomes: Our primary objective was to identify microbial signatures specifically associated with GD/GO by analyzing changes in microbial richness, evenness, and composition (see Appendix A). We excluded studies not directly exploring the microbiota.
▪ Study Type: We included cross-sectional, case–control, and prospective cohort studies while excluding conference reports, expert opinions, literature reviews, and case reports.
▪ Language: Studies published in English were included, while those in other languages were excluded.

### 2.3. Quality Assessment

We evaluated potential bias in each included study using tailored “study quality assessment tools” designed for Observational Cohort and Cross-Sectional Studies, as well as for Controlled Intervention Studies. These tools were developed by the National Heart, Lung, and Blood Institute at the National Institutes of Health [17]. Both researchers individually filled out the assessment forms, and any inconsistencies were addressed through discussion until a consensus was reached. Each possible risk criterion was assigned a point value ranging from 1 (indicating low risk) to 0 (indicating high risk). These values were then totaled and expressed as a percentage. Based on this percentage, we summarized the critical evaluation as follows: Quality Rating—Poor (<50%), Fair (50–75%), or Good (≥75%). Supplementary Tables S3 and S4 provide an overview of the quality ratings assigned to the various studies.

## 3. Results

### 3.1. Study Selection and Characteristics

Initial screening yielded 229 studies, from which 63 duplicates were removed. Among the remaining 166 studies, 110 were considered irrelevant, and three were not retrieved. This process left 53 full-text studies for eligibility assessment. Subsequently, 29 additional studies were excluded for various reasons, as illustrated in the PRISMA flowchart in Figure 1. Ultimately, 25 studies met the inclusion criteria, comprising 23 monocentric and two multicentric studies [18,19,20,21,22,23,24,25,26,27,28,29,30,31,32,33,34,35,36,37,38,39,40,41,42]. Table 1 depicts the characteristics of the included studies (a more extended version is available in Table S5 in the Supplementary Material). Geographically, the majority of the studies were conducted in China (21/25), with three in European countries, and one in Egypt. Of the included papers, 18 were case–control studies, one was a cross-sectional study, and six were prospective cohorts.

Microbiota data in GD were extracted from 19 studies involving 713 GD patients and 546 HCs. Similarly, data on microbiota in GO were obtained from eight studies, encompassing 356 GO patients and 187 controls. Predominantly, GD/GO patients were female and aged between 30 and 50 years. While all GD studies focused on analyzing gut microbiota through fecal samples, GO studies varied: five analyzed gut microbiota, two studied orbital microbiota (ocular surface or adipose tissues), and one examined microbiota from both sites. Taxonomic structure analysis of the microbiota was determined using the amplification of the 16S rRNA marker–gene approach in 22 studies, while Shotgun metagenomic sequencing was employed in three studies (see Table 2 and Table 3).

Tables S6 and S7 provide extended versions of Table 2 and Table 3, including sequencing methods, sampling sites, treatment conditions, and patient characteristics, and are available in the Supplementary Material. 

### 3.2. Alpha and Beta Diversities in GD/GO

Data regarding the analysis of microbiota in patients with GD are summarized in Table 3. Comparisons between GD patients and HCs were applicable in 16 studies. The gut microbiota richness and evenness were reduced in GD patients compared to controls in 10 out of 16 (62.5%) of the analyzed populations. Divergent findings were observed in six studies: two reported enrichment in alpha diversity (see definitions in Appendix A), two found similar microbial richness and abundances between GD subjects and controls, while two studies showed conflicting results in alpha diversity indices within the same samples.

In Table 3, focusing on the microbial characteristics associated with GO, two types of microflora were scrutinized: fecal and orbital. Among the eight studies incorporated, alpha diversity was directly evaluated in five studies, utilizing fecal samples in three studies, orbital tissue samples in one study, and ocular surface specimens in one study. In contrast to GD, there is no consistent pattern observed in gut microbiota richness and evenness in GO. One study reported an increase, another found similarity to HCs, and a third study could not draw conclusions due to conflicting alpha diversity indices. Similar inconclusive findings were noted in the orbital microbiota of GO patients: while one study suggested a reduction in alpha diversity in orbital adipose tissue, another found comparable microbial richness in ocular surface samples from GO and HC subjects.

Beta diversity (see definitions in Appendix A) was examined between GD and HC samples in 16 studies, and the results were more consistent, showing significant taxonomic divergence between microbial clusters in GD and HCs in 13 out of 16 (81.3%) publications. Two studies reported an overlap between the two groups, while in the remaining study, the overlap was only observed between samples of mild GD patients and HCs. However, a clear distinctive pattern was observed between patients with severe GD and HCs. As for GO, beta diversity analysis of samples from the intestine and orbital adipose tissues confirmed a clear separation in microflora composition between GO subjects and controls, except for ocular surface samples where the compositions overlapped.

### 3.3. Taxonomic Composition Associated with GD/GO

Phylum taxonomy related to GD/GO was examined in 18 out of 25 studies involving fecal samples from patients with GD/GO. The gut microbiota composition GD exhibits significant variability across studies, particularly at the genus level, as depicted in Table 3. The Firmicutes to Bacteroidetes ratio (F/B ratio) was consistently decreased in GD patients, with 10 out of 15 instances (66.7%) showing significant reductions. Conversely, this ratio was comparable between GD and controls in only two studies. Three authors reported a spontaneous increase in Firmicutes in GD patients compared to HCs.

In GO, the taxonomic analysis of microbiota provided in five studies revealed a tendency towards increased Bacteroidetes phyla and decreased Firmicutes in gut and orbital adipose microflora, as illustrated in Table 3. However, one study deviated from this trend and showed an inverse phylum pattern in gut microbiota. Another exception was observed in the microflora of ocular surfaces, which demonstrated an increase in different phyla, specifically Proteobacteria.

At the genus level, results were highly conflicting due to the heterogeneity of findings across the included studies. However, some genera appear to be closely associated with GD/GO. Genera that consistently showed an increase included *Prevotella* (11 studies), genera within the Lactobacillaceae family (three studies), and *Streptococcus* (three studies). Conversely, a general tendency to decrease was observed in genera from the families Lachnospiraceae (nine studies), Ruminococcaceae (six studies), and Veillonellaceae (five studies), as well as the genus *Bacteroides* (three studies). A concise overview of the taxonomic profiles associated with GD/GO is presented in the graphical abstract.

### 3.4. Association Between Host Microbiota and the Severity of GD/GO

Among the included studies, 10 papers statistically analyzed the impact of microbiota in hosts affected by GD/GO and parameters reflecting disease severity, such as clinical manifestations, Clinical Activity Score of GO, and TRAb levels. In one study, alpha diversity indices, specifically the Pielou and Simpson indices, were negatively correlated with TRAb levels. Additionally, in five studies, the genus *Lactobacillus* was significantly correlated with TRAb levels and/or the severity of GO. In contrast, a European multicentric study found that all genera uniquely associated with TRAb were Firmicutes of the Clostridiales family. In another study, the abundance of *Klebsiella pneumoniae* was significantly increased in the sight-threatening GO group.

### 3.5. Effect of ATDs on the Microbiota in GD/GO

ATD therapy significantly alters gut microbiota in GD but does not restore a profile comparable to HCs. Alpha diversity showed partial recovery after treatment (three studies), with increased richness and evenness compared to untreated GD, although remaining lower than HCs (two studies). A reduction in diversity following prolonged methimazole exposure was also reported (one study), whereas adjunctive interventions (probiotics, berberine) were associated with improved diversity (two studies). Beta diversity was consistently modified by ATD therapy (five studies), with treated patients exhibiting distinct clustering from both HC and baseline microbiota (three studies). Persistent separation from HCs in most studies (three studies) indicates incomplete microbial normalization.

At the taxonomic level, the F/B ratio increased after treatment in two studies but remained altered compared to HCs. *Faecalibacterium* decreased (2 studies), whereas *Eubacterium rectale* increased (1 study). Genera enriched in untreated GD, including *Lactobacillus*, *Veillonella*, and *Streptococcus*, tended to decline after treatment (one study), while *Phascolarctobacterium* increased (one study). Adjunctive strategies further modulated the microbiota, with probiotics and dietary supplementation improving composition and reducing TRAb levels (two studies).

In GO, data on ATD-related microbiota changes are limited and heterogeneous, and do not support restoration toward HC profiles. Alpha diversity remained reduced (two studies) or unchanged (one study), while β-diversity differences between treated GO and HC persisted (two studies).

At the taxonomic level, dysbiosis was maintained under ATD therapy, characterized by increased Bacteroidetes and decreased Firmicutes (two studies), leading to a reduced Firmicutes/Bacteroidetes ratio, along with increased Actinobacteria (one study). At the genus level, *Prevotella* and Prevotellaceae remained enriched (two studies) and correlated with TRAb levels, whereas short-chain fatty acid-producing genera such as *Blautia*, *Anaerostipes*, and *Butyricicoccus* remained reduced (two studies). Taxa associated with disease activity, including *Lactobacillus* and *Bifidobacterium*, continued to correlate positively with GO severity despite treatment (one study).

## 4. Discussion

Despite the high prevalence of GD and GO, prior investigations into the microbiome of individuals affected by hyperthyroidism have predominantly focused on thyroid diseases in general or autoimmune conditions overall. To our knowledge, this systematic review represents the most comprehensive effort to date, encompassing a total of 25 studies specifically aimed at elucidating the microbiota profile associated with GD/GO.

Early evidence suggested an association between GD and the gastrointestinal tract, supported by evidence such as gastrointestinal infections (e.g., *Yersinia enterocolitica*, *Helicobacter pylori*, Hepatitis C virus) contributing to GD pathogenesis through molecular mimicry [43,44]. Altered intestinal transit and the observed association between AITDs and other digestive conditions such as celiac disease and pernicious anemia further support this link [9]. The identification of a “thyroid–gut” axis, was facilitated thanks to murine models [9,45]. Experimental methods such as fecal microbiota transplantation emphasize how abnormal intestinal microbiota contribute to the susceptibility of mouse strains to GD and exacerbate disease progression [46,47].

In the context of GD, a consistently observed pattern is the reduced alpha diversity of gut microflora [18,21,22,23,26,29,31,36,38,39]. Su et al. established a negative correlation between Pielou and Simpson indexes and the serum levels of TRAb, FT3, and FT4, indicating a significant association with the intensity of GD [23]. Conversely, only three studies found no significant difference in alpha diversity between individuals with GD and healthy controls [25,27,28].

GO could involve changes not only in the microbiota along the thyroid–gut axis but also in the microflora of the orbit. The examination of microflora on the ocular surface or within hyperactive orbital adipose tissue has yielded varied findings compared to those reported in GD. While two studies noted decreased alpha diversity in fecal and orbital adipose tissue samples from individuals with GO compared to HCs [33,34,42], others found similar microbial diversities in fecal and ocular surface specimens between GO and HC groups. However, conflicting results were observed in the study by Shi et al., where the Shannon diversity index was reduced, yet no significant differences were detected in the Chao1 and ACE richness indexes between GD patients and controls [32]. These discrepancies may be attributed to the limited number of included patients and the heterogeneity of their thyroid status, with some receiving ATDs, others being euthyroid, and some remaining untreated.

In the human intestines, the predominant phyla typically include Firmicutes, Bacteroidetes, Actinobacteria, and Proteobacteria [15,48]. The F/B ratio serves as a widely accepted marker for detecting gut dysbiosis, as disruptions in the balance between these dominant gut phyla have been implicated in the development of various metabolic and autoimmune conditions [48,49,50]. Our comprehensive analysis highlights significant differences in the taxonomic composition of the gut microbiota between patients diagnosed with GD/GO and their healthy counterparts. Several authors have noted a discernible trend characterized by a decrease in Firmicutes and an increase in Bacteroidetes among fecal samples from individuals with GD/GO, reflecting an imbalance in the overall F/B ratio [18,19,23,27,28,29,31,32,38].

The Firmicutes phylum, which includes genera such as *Bacillus*, *Clostridium*, *Lactobacillus*, and *Ruminococcus*, is consistently altered in GD/GO, with a general depletion that may represent a recurrent microbial signature observed across studies [49,51]. This reduction is particularly evident within the Lachnospiraceae family, where multiple SCFA-producing genera—including *Ruminococcus*, *Blautia*, Lachnospiraceae_NK4A136_group, *Eubacterium rectale*_group, *Subdoligranulum*, and *Roseburia*—are consistently decreased in GD [18,27,31,36] and GO [19,20,32], including in orbital adipose tissue [41]. Given their role in SCFA production and immune homeostasis, their depletion may promote pro-inflammatory states implicated in GD/GO pathogenesis [51,52,53].

In contrast, *Lactobacillus* is repeatedly increased in GD/GO [10,22,31] and correlates with TRAb levels, disease severity, and orbital adipogenesis [10,33,41,42]. Although often considered beneficial, it may exert pro-inflammatory effects through cytokine induction (IL-6, TNF-α), and has been linked to autoimmune diseases such as Crohn’s disease and autoimmune hepatitis [54,55,56,57]. Experimental data further support a potential pathogenic role in GD/GO via exacerbation of TSHR-induced autoimmunity [46].

Within Firmicutes, the Veillonellaceae family also shows dysregulation, with increased *Veillonella* reported in GD [10,22,39], while *Dialister* is reduced in some cohorts [18,30,36,42], suggesting heterogeneous involvement in autoimmune processes.

A consistent and functionally relevant finding in our systematic review is the reduction in *Faecalibacterium* in GD/GO [24,26,27]. It is conceivable that the loss of this major SCFA-producing taxon—normally responsible for generating anti-inflammatory metabolites that inhibit NF-κB signaling [58,59], and restrain Th1/Th17 activity [60], both central to GD/GO immunopathology [43,61,62]—may contribute to a permissive environment for immune dysregulation and potentially facilitate NF-κB overactivation [63].

The Bacteroidetes phylum, particularly *Prevotella*, is markedly expanded in GD/GO, with 11 studies reporting increased abundance [18,19,22,23,27,28,31,32,36,37,40]. *Prevotella* correlates positively with TRAb levels [20], and has been implicated in Th17-mediated inflammatory diseases [64,65,66]. It is plausible that certain *Prevotella* strains, acting as pro-inflammatory pathobionts, could contribute to the initiation or amplification of GD by promoting Th17-driven immune responses. In contrast, *Bacteroides*—although generally reduced in GD/GO across several studies [18,27,41], with one report showing an increase [31]—may play a different immunomodulatory role. Its decreased abundance has been proposed as a potential biomarker of GO severity [41], and metabolomic evidence raises the possibility that *Bacteroides* influences Treg/Th17 balance through propionic acid-related pathways [23].

*Alistipes* is a relatively recent taxon primarily isolated from medical clinical samples, albeit at a lower frequency compared to other genera within the Bacteroidetes phylum. Some authors have proposed that this key SCFA-producing genus may play a role in suppressing Th17 cell activity within the gut, potentially contributing to a more regulated immune environment [67]. This role may even extend to mitigating inflammatory processes, as suggested by murine studies in which similar mechanisms appeared to lessen hepatic fibrosis and, in turn, seemed to limit cancer cell proliferation within liver tissue [68,69]. The observed decrease in *Alistipes*, as reported in two cohorts of GD patients, may potentially contribute to the pathogenesis of autoimmune hyperthyroidism through a comparable mechanism [18,24].

Moreover, an expansion of Proteobacteria is observed in GD/GO [18,21,23,37], reinforcing a dysbiotic pattern associated with inflammation. Within this phylum, *Klebsiella pneumoniae* has been linked to sight-threatening GO [40]. Ocular tissues in GO are predominantly colonized by several Proteobacteria genera (*Brevundimonas*, *Pseudomonas*, *Comamonas*, etc.), although their pathogenic role remains insufficiently defined [33,34]. However, because orbital adipose tissue sampling is highly susceptible to environmental and procedural contamination, it remains uncertain whether these detected taxa genuinely reflect resident ocular microbiota or merely represent incidental bacterial introduction during tissue handling.

ATD therapy clearly influences the gut microbiota in Graves’ disease GD, but it does not fully restore a healthy control-like ecosystem. Overall, it leads to a partial recovery of alpha diversity, which remains lower than in healthy controls [29,30]. It consistently reshapes community structure on β-diversity analyses, with treated patients still clustering separately from both baseline and controls [36]. At the phylum level, a persistent pattern of incomplete correction is observed, with only partial normalization of the F/B ratio, continued depletion of key SCFA-producing bacteria such as *Faecalibacterium*, and limited recovery of beneficial taxa like *Eubacterium rectale* [29,30]. Overall, ATD therapy appears to reshape the microbiota without fully reversing the underlying disease-associated dysbiosis. In contrast to GD, findings in GO are more heterogeneous and less consistent, reflecting smaller study numbers and variability in sampled sites and disease phenotypes.

## 5. Limitations

This systematic review acknowledges several limitations that warrant attention for a comprehensive contextualization of the findings. The significant shortcoming arises from the predominant geographical bias, with 84% of the studies conducted in China. Dietary patterns, lifestyle factors, and environmental exposures in Asian populations markedly differ from Western and Mediterranean diets, potentially skewing the microbial landscape. This geographical concentration restricts the generalizability of the identified microbial signature in GD/GO to broader populations.

Additionally, limited sample sizes and the use of heterogeneous sequencing platforms may potentially compromise reproducibility in statistical analyses and conclusions reported. Regrettably, several observational studies have concentrated solely on characterizing gut/orbital microbiota. This has impeded the establishment of causal relationships between gut microbiota and GD/GO. A comprehensive assessment of intermediate metabolites and biological pathways is essential for a nuanced understanding beyond the variation of specific bacterial strains.

## 6. Conclusions

Our systematic review provides a comprehensive compilation of current evidence about the relationship between host microbiota and GD/GO. GD/GO-associated dysbiosis is characterized by reduced microbial richness and evenness and also manifests in alterations of gut phyla proportions, including a decrease in Firmicutes, an increase in Bacteroidetes, and a rise in Proteobacteria. Specific genera such as *Lactobacillus*, *Prevotella*, *Bacteroides*, and members of the Lachnospiraceae family may be involved in the initiation or progression of GD/GO through potential effects on the Th17/Treg balance, although their exact roles remain uncertain and require further clarification.

While current therapeutic approaches for GD and GO, such as ATDs, corticosteroids, and immunosuppressants, primarily address the consequences of the autoimmune reaction, microbiota-targeting therapeutics may represent a potential avenue for addressing some of the underlying factors associated with GD/GO.

Further investigation, including studies with larger sample sizes and participants from diverse geographical backgrounds, is essential to elucidate the distinct mechanisms underlying the thyroid–gut axis in both health and disease. Additionally, carefully designed clinical trials are needed to evaluate the effects of pre and probiotics on this axis and their impact on GD/GO outcomes.

## Figures and Tables

**Table 1:** Characteristics of included studies investigating dysbiosis in Graves’ disease and/or Graves’ ophthalmopathy.

Authors	Country	Study Design	Microbiota Site	Sample Size	Main Objective
Ishaq et al. [18]	Pakistan and China (2018)	Case–control	Fecal	27 GD vs. 11 HC	Evaluate the diversity and similarity of intestinal microbiota qualitatively and quantitatively in GD as compared to their healthy counterparts.
Shi et al. [19]	China (2019a)	Case–control	Fecal	33 GO vs. 32 HC	Investigate whether GO patients differ from healthy controls in the fecal microbiota.
Shi et al. [20]	China (2019b)	Cross-sectional	Fecal	31 GO	Explore the relationships between gut microbiota and GO-related traits.
Yang et al. [21]	China (2019)	Case–control	Fecal	15 GD vs. 15 HC	Explore the association of intestinal flora alteration with the development of GD among the Han population in southwest China.
Yan et al. [22]	China (2020)	Case–control	Fecal	39 GD vs. 07 HC	Investigate changes in intestinal flora that may occur in the setting of GD.
Su et al. [23]	China (2020)	Case–control	Fecal	63 GD vs. 58 HC	Investigate the association and mechanism between intestinal flora and GD.
Sun et al. [24]	China (2020)	Prospective	Fecal	40 GD vs. 50 HC	Observe changes in the gut microbiota structure caused by ATDs.
Cornejo-Pareja et al. [25]	Spain (2020)	Case–control	Fecal	09 GD vs. 11 HC	Investigate the possible relationship between gut microbiota composition and the most frequent AITDs.
Zhu et al. [26]	China (2021)	Case–control	Fecal	100 GD (36 Mild GD + 64 Severe GD) vs 62 HC	Describe the intestinal microbial characteristics and microbial mutations of GD patients.
Chang et al. [27]	China (2021)	Case–control	Fecal	55 GD vs. 48 HC	Characterize the composition of gut microbiota in GD patients.
El-Zawawy et al. [28]	Egypt (2021)	Case–control	Fecal	13 GD vs. 30 HC	Elucidate changes in gut microbiome in Egyptian patients with ATDs.
Chen et al. [29]	China (2021)	Prospective	Fecal	15 GD vs. 14 HC	Investigate the correlation between human gut microbiota and clinical characteristics and thyroidal functional status of GD.
Huo et al. [30]	China (2021)	Prospective	Fecal	26 GD: 08 treated with MMI 09 treated with (MMI + black-bean) 09 treated with (MMI + probiotic *Bifidobacterium longum*)	Evaluate the curative effects of probiotics supplied with MI on thyroid function of patients with GD.
Jiang et al. [31]	China (2021)	Case–control	Fecal	45 GD vs. 59 HC	Examine the makeup and metabolic function of microbiota in GD patients.
Shi et al. [32]	China (2021)	Case–control	Fecal	30 GD vs. 33 GO vs. 32 HC	Identify specific intestinal bacteria of GD and GO, respectively.
Ji et al. [33]	China (2022)	Case–control	Ocular surface	67 GO vs. 22 HC	Investigate the diversity and composition of the ocular microbiota in patients with GO.
Li et al. [34]	China (2022)	Case–control	Orbital adipose tissue	27 GO vs. 27 HC	Investigate whether bacteria were present in the orbital adipose tissue of subjects with GO and if the amount and composition of these bacteria were correlated with the disease phenotypes.
Han et al. [35]	China (2022)	Prospective	Fecal	08 GD treated with MMI 10 GD treated with MMI + Berberine	Explore the mechanism by which the combination of MMI and berberine may regulate the intestinal microbiota of patients with GD.
Yang et al. [36]	China (2022)	Case–control	Fecal	18 Untreated GD vs. 10 Treated GD vs. 11 HC	Analyze the relationships between changes in the intestinal flora, thyroid function, and relevant thyroid antibodies in GD patients before and after MMI treatment.
Zhao et al. [37]	China (2022)	Case–control	Fecal	27 GD vs. 16 HC	Explore the role of gut microbiota in GD and HT.
Jiang et al. [38]	China (2023)	Case–control	Fecal	39 GD vs. 48 HC	Identify specific microbiota and metabolites that could distinguish Graves’ disease patients, hypothyroidism patients, and controls.
Deng et al. [39]	China (2023)	Prospective	Fecal	65 GD vs. 33 HC	Profile the gut microbiota of patients newly diagnosed with GD before and after treatment.
Zhang et al. [40]	China (2023)	Case–control	Fecal	62 GO: (20 mild, 25 moderate, 17 severe) vs. 18HC	Explore the changes of gut microbiota in GO patients of different severity grades.
Biscarini et al. [41]	UK, Italy, Belgium, and Germany (2023)	Prospective	Fecal	59 GD vs. 46 GO vs. 41 HC	Compare the fecal microbiota in GD patients, with GO of varying severity, and HCs.
Fenneman et al. [42]	Netherlands (2023)	Case–control	Fecal Orbital adipose tissue samples (for operated GO)	57 GO (42 non-operated + 15 operated) vs. 15 HC (operated)	Evaluate the hypothesis stating that enhanced intestinal permeability may aggravate orbital inflammation.

ATD: Antithyroid Drugs; GD: Graves’ Disease; GO: Graves’ Ophthalmopathy; HC: Healthy Controls; HT: Hashimoto Thyroiditis; MMI: Methimazole.

**Table 2:** Gut Dysbiosis in Patients with Graves’ Disease: Alpha and Beta Diversities, and Taxonomic Composition.

1st Autor	Alpha Diversity	Beta Diversity	Taxonomic Composition		Additional Findings
			Phylum Level	Genus Level	
Ishaq et al. (2018) [18]	↓ α-diversity in GD.	Distinct microbiota structure from HCs.	F/B ratio ↓ in GD vs. HCs. Bacteroidetes ↑, Actinobacteria ↑, Proteobacteria ↑; Firmicutes ↓ in GD vs. HCs.	*Prevotella_9* ↑, *Haemophilus* ↑ in GD vs. HCs. *Bacteroides* ↓, *Ruminococcus* ↓, *Dialister* ↓, *Alistipes* ↓ in GD vs. HCs.	
Yang et al. (2019) [21]	↓ α-diversity indices in GD.	Separated microbiota structure from HCs.	F/B ratio ↑ in GD vs. HCs. Firmicutes ↑, Proteobacteria ↑, *Actinobacillus* ↑ in GD vs. HCs.	*Oribacterium* ↑, *Mogibacterium* ↑, *Lactobacillus* ↑, and *Aggregatibacter* ↑ in GD vs. HCs. *Prevotella* ↑ (non-significant) in GD vs. HCs.	
Yan et al. (2020) [22]	↓ richness and ↓ evenness in GD.	Distinct flora clustering from HCs.	N/D	Bacilli ↑, Lactobacillales ↑; *Prevotella* ↑, *Megamonas* ↑, and *Veillonella* ↑ in GD vs. HCs. *Ruminococcus* ↓, Rikenellaceae ↓, *Alistipes* ↓ in GD vs. HCs.	
Su et al. (2020) [23]	↓ α-diversity in GD.	Distinct microbiota composition from HCs.	F/B ratio ↓ in GD vs. HCs. Proteobacteria ↑, Saccharibacteria ↑, Verrucomicrobia ↑ in GD vs. HCs.	The random forest analysis also showed that 3 intestinal bacteria (*Bacteroides*, *Alistipes*, *Prevotella*) could distinguish GD patients from HCs with 85% accuracy. *Yersinia enterocolitica* significantly ↑ in GD patients with diarrhea than in GD patients without diarrhea and HCs.	The Pielou and Simpson indexes were significantly negative with the intensity of the disease.
Sun et al. (2020) [24]	Richness ↑ in GD. Richness ↑ after ATDs. Evenness ↑ after ATDs (but still < HCs).	β-diversity deviation of GD from HCs. Greater post-ATD deviation from HCs. Partial deviation from baseline after treatment.	F/B ratio ↑ pre-ATD. F/B ratio ↑ in treated GD vs. HCs.	*Faecalibacterium* ↓, *Clostridium*_sensu_stricto_1 ↓, *Eubacterium_rectale* ↑, *Romboutsia* ↑, and *Dorea* ↑ after ATDs.	ATD altered gut microbiota structure.
Cornejo-Pareja et al. (2020) [25]	The bacterial richness was comparable between GD and HC groups. Evenness ↓ in GD patients	Gut microbiota from both groups was different.	No significant difference in the F/B ratio between the two groups.	*Fusobacterium* ↑, *Faecalibacterium* ↓ in GD vs. HCs. The *Prevotella* genus seemed to be characteristic of the GD group.	TRAb level positively correlated with *Lactobacillus* and Pasteurellaceae. TRAb level negatively correlated with *Faecalibacterium*.
Zhu et al. (2021) [26]	α-diversity ↓ in severe GD.	Intestinal microbiota in the HC and mild GD groups were similar. In severe GD: distinct separation.	N/D	In all GD: *Coprobacillus* ↑, *Streptococcus* ↑, *Rothia* ↑. In severe GD: *Faecalibacterium*_*prausnitzii* ↓, *Butyricimonas*_*faecalis* ↓, *Bifidobacterium*_*adolescentis* ↓, *Akkermansia*_*muciniphila* ↓.	
Chang et al. (2021) [27]	The microbial richness and evenness of the GD group were similar to that of the HCs.	The overall community structure was distinctive between the two sample groups.	F/B ratio ↓ in GD vs. HCs. Bacteroidetes ↑,Actinobacteria ↑, Firmicutes ↓ in GD vs. HCs.	*Bacteroides* ↑, *Prevotella*_9 ↑, *Faecalibacterium* ↓, Lachnospiraceae_NK4A136_group ↓ in GD vs. HCs.	
El-Zawawy et al. (2021) [28]	No significant difference in α diversity was observed between the two groups.	The similarity in gut microbiota between GD and HC group was 68%.	F/B ratio ↓ in GD vs. HCs.	*Prevotella* ↑ in GD vs. HCs.	Significant positive TRAb correlation with Bacteroidetes.
Chen et al. (2021) [29]	Abundance ↓ and diversity ↓ in GD vs. HCs. Abundance ↑ and diversity ↑ after treatment.	Distinct composition and structure in GD vs. HCs.	F/B ratio ↓ in untreated GD vs. HCs. Proteobacteria ↓ and Synergistetes ↓ in GD vs. HCs. Proteobacteria ↑ after ATDs vs. pre-ATDs.	*Lactobacillus* ↑, *Veillonella* ↑, *Streptococcus* ↑ in GD vs. HCs. Post-ATD changes: *Blautia* ↓, Corynebacter ↓, *Ruminococcus* ↓, *Streptococcus* ↓; *Phascolarctobacterium* ↑.	Positive TRAb correlation with *Lactobacillus* and *Ruminococcus*. Negative TRAb correlation with Synergistetes and *Phascolarctobacterium*.
Huo et al. (2021) [30]	Microbial α-diversity ↓ after 6-month MMI vs. baseline.	MMI-induced microbiota alteration in GD. Black-bean adjuvant MMI maintain microbiome homeostasis during 6-month treatment.	Not studied	*Faecalibacterium prausnitzii* ↓, *Ligilactobacillus salivarius* ↓, *Lactococcus lactis* ↓, *Porphyromonas* spp. ↓, *Prevotella* spp. ↓ in patients treated with MMI.	*Bifidobacterium longum* adjuvant MMI → improved thyroid function and TRAb ↓.
Jiang et al. (2021) [31]	Diversity ↓ and abundances of specific taxa ↓ in GD vs. HCs.	Microbial composition distinct in GD vs. HCs.	F/B ratio ↓ in untreated GD vs. HCs.	*Faecalibacterium* ↑, *Bacteroides* ↑, *Prevotella*_9 ↑, *Bifidobacterium* ↑, *Blautia* ↓, *Subdoligranulum* ↓, [*Eubacterium*]_*rectale*_group ↓ in GD vs. HCs.	*Bacteroides*, *Blautia*, [*Eubacterium*]_*hallii*_group, *Anaerostipes*, *Lactobacillus*, *Dorea* could serve as diagnostic biomarkers of GD.
Han et al. (2022) [35]	α-diversity ↓ in MMI-treated GD patients. α-diversity ↑ after MMI treatment supplemented with berberine.	The addition of berberine reshaped the structure of the patients’ gut microbiota in contrast to MMI alone.	N/D	MMI alone failed to modulate the gut microbiota of the patients. Microbiota shift after MMI + berberine: *Lactococcus lactis* ↑, *Enterobacter hormaechei* ↓, *Chryseobacterium indologenes* ↓, and *Prevotella* spp. ↓.	
Yang et al. (2022) [36]	Intestinal diversity ↓ in GD vs. HCs. Diversity in untreated GD > treated GD.	Partial overlap among HCs, untreated GD, and treated GD.	F/B ratio ↓ in GD vs. HCs. Actinobacteria ↑, Cyanobacteria ↑, TM7 ↑; Firmicutes ↓, [Thermi] ↓ in untreated GD vs. HCs. Proteobacteria ↑, TM7 ↑; [Thermi] ↓ in treated GD vs. HCs. Actinobacteria ↑ in untreated GD vs. treated GD.	*Collinsella* ↑ in untreated GD vs. HCs and treated GD. *Bifidobacterium* ↑; *Dialister* ↓; *Roseburia* ↓ in untreated GD vs. HCs. *Prevotella* ↓ in treated GD vs. HCs and untreated GD.	
Zhao et al. (2022) [37]	α-diversity ↑ in GD vs. HCs.	Gut microbiota significantly different between the GD group and HC group.	Proteobacteria ↑, Firmicutes ↑, Cyanobacteria ↑ in GD vs. HCs.	*Prevotella*_9 ↑, *Ruminococcus*_2 ↑, Lachnospiraceae_NK4A136_group ↑ in GD vs. HCs.	*Bacillus*, *Blautia*, and Ornithinimicrobium could be used as potential markers of GD.
Jiang et al. (2023) [38]	α-diversity ↓ in GD vs. HCs.	Significant distinction in microbial composition between GD and HC groups.	F/B ratio ↓ in GD vs. HCs.	*Bacteroides* ↑, *Lactobacillus* ↑, *Blautia* ↓, [*Eubacterium*]_*hallii*_group ↓, *Collinsella* ↓ in GD vs. HCs.	
Deng et al. (2023) [39]	Richness ↓ and evenness ↓ in GD vs. HCs.	Gut microbiota composition of patients with GD was significantly different from that of HCs.	No significant difference in the F/B ratio in GD vs. HCs.	*Streptococcus* ↑, *Veillonella* ↑, *Erysipelatoclostridium* ↑, *Roseburia* ↓, *Romboutsia* ↓, Lachnospira ↓, *Eubacterium ventriosum* ↓ in GD vs. HCs.	Gradual microbiota reconstruction and recovery of the intestinal flora in GD after ATD treatment.

↑: Increase; ↓: Decrease; ATD: Antithyroid drug; GD: Graves’ disease; HCs: Healthy controls; MMI: Methimazole; N/D: Not determined; TRAb: Thyrotropin receptor antibody.

**Table 3:** Gut and orbital dysbiosis in patients with Graves’ orbitopathy: alpha and beta diversities, and taxonomic composition.

Authors	Site	Alpha Diversity	Beta Diversity	Taxonomic Composition		Additional Findings
				Phylum Level	Genus Level	
Shi et al. (2019a) [19]	Fecal	α-diversity ↓ in GO vs. HCs.	Significant separation in fecal microbiota between GO patients and HCs.	Bacteroidetes ↑ and Firmicutes ↓ in GO vs. HCs.	Prevotellaceae ↑, *Blautia* ↓, Fusicatenibacter ↓, *Butyricicoccus* ↓, *Anaerostipes* ↓, and *Collinsella* ↓ in GO vs. HCs.	Positive correlation of TRAb with Succinivibrionaceae.
Shi et al. (2019b) [20]	Fecal	N/D	Not applicable	Firmicutes and Bacteroidetes: most predominant phyla in GO patients. Bacteroidetes: very high proportion among top OTUs.	N/D	The genera, s_*Prevotella*_*copri* and f_Prevotellaceae, showed a significant positive correlation with TRAb.
Shi et al. (2021) [32]	Fecal	Shannon ↓ in GO vs. HCs. Chao1 = no difference. ACE = no difference.	Clear separation in intestinal bacteria in GO vs. HCs.	F/B ratio ↓ in GO vs. HCs. Bacteroidetes ↑ and Firmicutes ↓ in GO vs. HCs.	Unidentified_Prevotellaceae ↑, *Blautia* ↓, Fusicatenibacter ↓, *Butyricicoccus* ↓, *Anaerostipes* ↓, *Collinsella* ↓ in GO vs. HCs. *Subdoligranulum* ↑, *Bilophila* ↑, Blautia ↓, *Anaerostipes* ↓, *Dorea* ↓, *Butyricicoccus* ↓, *Romboutsia* ↓, Fusicatenibacter ↓, unidentified_Lachnospiraceae ↓, unidentified_Clostridiales ↓, *Collinsella* ↓, *Intestinibacter* ↓, and *Phascolarctobacterium* ↓ in GO vs. GD	
Ji et al. (2022) [33]	Ocular surface	No significant difference in α diversity between the GO vs. HCs.	No significant aggregation difference between the two groups	Dominant phyla (same order in both groups): Proteobacteria > Firmicutes > Actinobacteria > Bacteroidetes. Proteobacteria ↑, Firmicutes ↓, Acidobacteriota ↑, Verrucomicrobiota ↑, and Actinobacteria ↓ in GO vs. HCs.	*Bacillus* ↑, *Brevundimonas* ↑, and *Corynebacterium* ↓ in GO vs. HCs.	*Paracoccus*, *Haemophilus*, *Lactobacillus*, and *Bifidobacterium* positively correlated with the severity of clinical manifestations or disease activity.
Li et al. (2022) [34]	Orbital adipose tissue	GO orbital fat microbiota diversity ↓ vs. HCs.	Significant differences between GO patients and HCs.	Bacteroidetes ↑ and Firmicutes ↓ in GO vs. HCs.	*Pseudomonas* ↑, *Comamonas* ↑, *Brevundimonas* ↑, Aeromonas ↑, *Flavobacterium* ↑, and *Janthinobacterium* ↑ in GO vs. HCs.	
Zhang et al. (2023) [40]	Fecal	No significant difference in gut microbiota α diversity between the groups.	The gut microbial community between the control and GO groups differed significantly.	No F/B ratio across four groups.	*Faecalibacterium prausnitzii* ↑ in moderate–severe vs. mild GO. *Klebsiella pneumoniae* ↑ in sight-threatening GO.	*Klebsiella_pneumoniae* was a potential GO-related pathogen, which may regulate the metabolic pathways to affect GO progression.
Biscarini et al. (2023) [41]	Fecal	N/D	N/D	F/B ratio ↑ in GO vs. HCs and ↑ in all GD/GO cases. Actinobacteria ↑ in GD and GO vs. HCs; GO > GD. Bacteroidetes ↓ in GD and GO vs. HCs.	Mild GO: *Bacteroides* spp. ↓, *Bifidobacterium* spp. ↑, *Fusicatenibacter* spp. ↑. Moderate–severe GO: *Roseburia* spp. ↑ vs. HCs, GD, and mild GO.	*Bacteroides* spp. represented one of the top bacterial biomarkers when predicting GO severity. All genera uniquely associated with TRAb were Firmicutes of the Clostridiales family.
Fenneman et al. (2023) [42]	Fecal Orbital adipose tissue samples	N/D	N/D	N/D	*Bacteroides* spp. and *Dialister* spp., were positively correlated with the concentration of serum lipopolysaccharide-binding protein, linking the gut to local orbital inflammation.	*Lactobacillus* abundance in stool samples was shown to be associated with the severity of GO and specifically with orbital adipogenesis.

↑: Increase; ↓: Decrease; ACE: Abundance-based coverage estimator; GO: Graves’ orbitopathy; HCs: Healthy controls; N/D: Not determined; OTU: Operational taxonomic unit; TRAb: Thyrotropin receptor antibody.

## Data Availability

No new data were created in this study.

## Supplementary Materials

The following supporting information can be downloaded at: https://www.mdpi.com/article/10.3390/jcm15124586/s1: Table S1: PRISMA 2020 Checklist; Table S2: Eligibility Criteria for Studies Analyzing Microbiota in Graves’ Disease and Graves’ Orbitopathy (PICOS Framework Approach); Table S3. Quality Assessment Tool of Controlled Intervention Studies; Table S4. Quality Assessment Tool for Observational Cohort and Cross-Sectional Studies; Table S5: Characteristics of Included Studies Investigating Dysbiosis in Graves’ Disease and/or Graves’ Ophthalmopathy (extended version); Table S6: Gut Dysbiosis in Patients with Graves’ Disease: Alpha and Beta Diversities, and Taxonomic Composition (extended version); Table S7: Gut and Orbital Dysbiosis in Patients with Graves’ Orbitopathy: Alpha and Beta Diversities, and Taxonomic Composition (extended version).

## Abbreviations

The following abbreviations are used in this manuscript:

AITDs	Autoimmune Thyroid Disorders
ATD	Antithyroid Drugs
EUGOGO	European Group on Graves’ Orbitopathy
F/B ratio	*Firmicutes* to *Bacteroidetes* ratio
GD	Graves’ Disease
GO	Graves’ Orbitopathy
HC	Healthy Control
IBD	Inflammatory Bowel Disease
MMI	Methimazole
PICO	Population, Intervention, Comparison, and Outcome
PRISMA	Preferred Reporting Items for Systematic Reviews and Meta-Analyses
PTU	Propylthiouracil
SCFAs	Short-Chain Fatty Acids
TRAb	Thyroid Receptor Autoantibodies

## Appendix A. Key Microbiome-Related Terminology in Human Health and Disease

▪ **Microbiome**: The microbiome is the collective set of genomes from microorganisms (including bacteria, fungi, viruses, and archaea) that inhabit a particular environment, such as the human body. It not only includes the microorganisms themselves but also their genetic material, functioning as an ecological system that interacts with the host and influences various physiological processes.
▪ **Microbiota**: The microbiota refers to the community of microorganisms, including bacteria, fungi, viruses, and other microbes, that live in a specific environment, such as the gut, skin, or oral cavity. It is often examined in terms of its composition, diversity, and its impact on health and disease.
▪ **Microbiota Taxonomy** refers to the classification system used to organize microorganisms within a microbiota, helping to categorize their diversity and relationships. It follows a hierarchical structure, with key taxonomic levels including: Domain (e.g., Bacteria, Archaea) ⮞ Phylum (e.g., Firmicutes, Proteobacteria) ⮞ Class (e.g., Clostridia, Gamma-proteobacteria) ⮞ Order (e.g., Lactobacillales) ⮞ Family (e.g., Lactobacillaceae) ⮞ Genus (e.g., *Lactobacillus*, *Bacteroides*) ⮞ Species (e.g., *Lactobacillus acidophilus*, *Bacteroides fragilis*).
▪ **Dysbiosis**: This term is most commonly used to describe an overall disruption in the microbiota’s composition. This can involve alterations, disturbances, abnormal composition, or a reduction in diversity. Dysbiosis is typically associated with an imbalance in microbial communities, which is generally considered harmful, and may also refer to changes in the relative abundance of specific genera within the microbiota.
▪ **Alpha diversity** refers to the diversity of microorganisms within a single microbial community, such as in the human gut or skin. It is measured by the number of species (richness) and how evenly they are distributed (evenness). Higher alpha diversity is typically associated with better health, while lower diversity may indicate dysbiosis or disease.
▪ **Beta diversity** refers to the comparison of microbial diversity between different environments or samples, such as patients with Graves’ disease and non-healthy individuals. It measures the differences in species composition between these groups, reflecting how similar or distinct their microbial communities are.
▪ **Shannon Diversity Index**: A measure of both the richness and evenness of species in a community, with higher values indicating greater diversity.
▪ **Chao1 Diversity**: An estimator of species richness that accounts for rare or undetected species, providing a more accurate count of total species in a community.
▪ **ACE Richness**: A measure of species richness that, like Chao1, estimates the total number of species in a community, particularly by considering rare species.
▪ **Pielou’s Evenness Index:** A measure of how evenly the species are distributed in a community, with values closer to 1 indicating more even distribution.
▪ **Simpson Index**: A measure of diversity that accounts for both richness and the relative abundance of species, with lower values indicating higher diversity.
