# Supplementary material for: Gut and Orbital Dysbiosis Associated with Graves’ Disease and Graves’ Orbitopathy: A Systematic Review
Source: J Clin Med. 2026 Jun 12;15(12):4586. doi: 10.3390/jcm15124586 (PMC13301356; doi:10.3390/jcm15124586)
Supplement: Supplementary file 1 [file jcm-15-04586-s001.zip › Table S2.pdf]

Table S2: Eligibility Criteria for Studies Analyzing Microbiota in Graves' Disease and Graves' Orbitopathy (PICOS Framework Approach)

Aspects	Inclusion criteria	Exclusion criteria
**Population**	– Human adult patients aged > 18 years<br>– Patients diagnosed with GD according to conventional diagnostic criteria, including clinical and biochemical hyperthyroidism, characteristic goiter (confirmed clinically, or using ultrasound or scintigraphy), and elevated TRAb<br>– Patients diagnosed with GO according to EUGOGO criteria (in effect at the time of the study)	– Animal studies<br>– Children and adolescents aged < 18 years<br>– Pregnant or breastfeeding women<br>– Patients with thyroid diseases other than GD, including Hashimoto's thyroiditis and non-autoimmune hyperthyroidism<br>– Patients with orbital space-occupying diseases such as tumors or extraocular myositis
**Intervention**	– Newly diagnosed untreated patients (no intervention)<br>– Newly diagnosed patients treated with ATD or a combination of ATD and probiotics	– Medications that could affect the gut or orbital microbiota, including antibiotics, corticosteroids (systemic use or in eye drop), immunosuppressants, laxatives, proton pump inhibitors, probiotics, prebiotics, or symbiotics before inclusion in the study, Chinese herbal medicine, hormone substitution other than levothyroxine, antidiabetic drugs including insulin, metformin, acarbose, sulfonylurea, and wearing contact lenses.
**Comparison**	– Healthy controls of similar age, sex, and BMI<br>– Other patients with different severity grades of GD/GO	– History of conditions potentially altering the microbiota, including other autoimmune diseases (Type 1 diabetes, systemic lupus erythematosus, rheumatoid arthritis, autoimmune hepatitis,…) and metabolic diseases (obesity, Type 2 diabetes, …)<br>– Conditions directly affecting the digestive tract, including acute diarrhea, digestive surgery, irritable bowel syndrome, and inflammatory bowel disease, …<br>– Severe diseases (acute infections, stroke, heart disease, renal or hepatic dysfunction, cancer, …<br>– Extreme diets (vegan, carnivore, keto) and substance abuse (excessive smoking, alcohol, drugs,)
**Outcomes**	– Identifying microbial signature associated with GD/GO through analysis of modifications in microbial richness, evenness, and composition	– Studies that do not directly explore the microbiota, including those exclusively exploring metabolites or other factors
**Study type**	– Cross-sectional studies<br>– Case-control studies<br>– Prospective cohort studies	– Conference reports, Expert opinions, Literature reviews, Letters to the editor, Case reports and Corrigenda papers
**Language**	– English language	– Studies in other languages other than English

ATD: Antithyroid Drugs; BMI: Body Mass Index; EUGOGO: European Group on Graves Orbitopathy; GD: Graves' Disease; GO: Graves Orbitopathy; TRAb: Thyroid Receptor Antibodies
