# Supplementary material for: Gut and Orbital Dysbiosis Associated with Graves’ Disease and Graves’ Orbitopathy: A Systematic Review
Source: J Clin Med. 2026 Jun 12;15(12):4586. doi: 10.3390/jcm15124586 (PMC13301356; doi:10.3390/jcm15124586)
Supplement: Supplementary file 1 [file jcm-15-04586-s001.zip › Table S3.pdf]

Table S3. Quality Assessment Tool of Controlled Intervention Studies

Country (Year)	Author	Q1	Q2	Q3	Q4	Q5	Q6	Q7	Q8	Q9	Q10	Q11	Q12	Q13	Q14	Total Score	Quality Rating
China(2020)	Sun et al. [24]	Y	N	NR	N	N	N	Y	Y	NR	Y	Y	N	Y	Y	7/14 (50%)	Fair
China (2021)	Chen et al. [29]	N	NA	NA	NA	NA	Y	N	Y	NR	Y	Y	Y	Y	Y	7/10 (70%)	Fair
China (2021)	Huo et al. [30]	N	NA	NA	NA	NA	NR	NR	NR	NR	Y	N	Y	NA	Y	3/9 (33.3%)	Poor
China (2022)	Han et al. [35]	N	NA	N	N	N	NR	NR	NR	NR	NR	N	Y	NA	Y	2/12 (16.6%)	Poor
China (2023)	Deng et al. [39]	N	NA	N	N	N	Y	N	NA	NR	Y	Y	N	N	Y	4/12 (33.3%)	Poor
UK, Italy, Belgium, and Germany (2023)	Biscarini et al. [41]	N	NA	NA	NA	NA	N	N	NA	NA	Y	N	N	NA	Y	2/7 (28.5%)	Poor

Quality of included studies was assessed using the National Institutes of Health (NIH) Quality Assessment of Controlled Intervention Studies (www.nhlbi.nih.gov/health-pro/guidelines/in-develop/cardiovascular-risk-reduction/tools/). **1**. Was the study described as randomized, a randomized trial, a randomized clinical trial, or an RCT? **2**. Was the method of randomization adequate (i.e., use of randomly generated assignment)? **3**. Was the treatment allocation concealed (so that assignments could not be predicted)? **4**. Were study participants and providers blinded to treatment group assignment? **5**. Were the people assessing the outcomes blinded to the participants' group assignments? **6**. Were the groups similar at baseline on important characteristics that could affect outcomes (e.g., demographics, risk factors, co-morbid conditions)? **7**. Was the overall drop-out rate from the study at endpoint 20% or lower of the number allocated to treatment? **8**. Was the differential drop-out rate (between treatment groups) at endpoint 15 percentage points or lower? **9**. Was there high adherence to the intervention protocols for each treatment group? **10**. Were other interventions avoided or similar in the groups (e.g., similar background treatments)? **11**. Were outcomes assessed using valid and reliable measures, implemented consistently across all study participants? **12**. Did the authors report that the sample size was sufficiently large to be able to detect a difference in the main outcome between groups with at least 80% power? **13**. Were outcomes reported or subgroups analyzed prespecified (i.e., identified before analyses were conducted)? **14**. Were all randomized participants analyzed in the group to which they were originally assigned, i.e., did they use an intention-to-treat analysis?

Total Score: Number of yes; **NA,** not applicable; **NR,** not reported; **N,** no; **Y,** yes.

Quality Rating: Poor <50%, Fair 50-75%, Good ≥75%
