# Supplementary material for: Gut and Orbital Dysbiosis Associated with Graves’ Disease and Graves’ Orbitopathy: A Systematic Review
Source: J Clin Med. 2026 Jun 12;15(12):4586. doi: 10.3390/jcm15124586 (PMC13301356; doi:10.3390/jcm15124586)
Supplement: Supplementary file 1 [file jcm-15-04586-s001.zip › Table S4.pdf]

Table S4. Quality Assessment Tool for Observational Cohort and Cross-Sectional Studies

Country (Year)	Author	Q1	Q2	Q3	Q4	Q5	Q6	Q7	Q8	Q9	Q10	Q11	Q12	Q13	Q14	Total Score	Quality Rating
Pakistan and China (2018)	Ishaq et al. [18]	Y	N	NR	Y	N	NA	NR	N	N	N	Y	NR	NA	N	3/12 (25%)	Poor
China (2019a)	Shi et al. [19]	Y	Y	NR	Y	Y	NA	NR	Y	Y	N	Y	NR	NA	Y	8/12 (66.7%)	Fair
China (2019b)	Shi el al. [20]	Y	Y	NR	Y	N	NA	NR	Y	Y	N	Y	NR	NA	N	6/12 (50%)	Fair
China (2019)	Yang et al. [21]	Y	Y	NR	Y	N	NA	NR	N	N	N	Y	NR	NA	N	4/12 (33.3%)	Poor
China (2020)	Yan et al. [22]	Y	Y	NR	Y	N	NA	Y	N	Y	N	Y	NR	NA	N	6/12 (50%)	Fair
China (2020)	Su et al. [23]	Y	Y	NR	NR	N	Y	Y	Y	Y	Y	Y	NR	N	N	8/14 (57.1%)	Fair
Spain (2020)	Cornejo-Pareja et al. [25]	Y	Y	NR	Y	N	NA	NR	Y	Y	N	Y	NR	NA	N	6/12 (50%)	Fair
China (2021)	Zhu et al. [26]	Y	Y	NR	Y	N	NA	NR	Y	Y	N	Y	NR	NA	N	6/12 (50%)	Fair
China (2021)	Chang et al. [27]	Y	Y	NR	Y	N	NA	Y	Y	Y	N	Y	NR	NA	N	7/12 (58.3%)	Fair
Egypt (2021)	El-Zawawy et al. [28]	Y	Y	NR	Y	N	NA	NR	Y	Y	N	Y	NR	NA	NR	6/12 (50%)	Fair
China (2021)	Jiang et al. [31]	Y	Y	NR	Y	N	NA	NR	Y	Y	N	Y	NR	NA	N	6/12 (50%)	Fair
China (2021)	Shi et al. [32]	Y	Y	NR	N	N	NA	NR	Y	Y	N	Y	NR	NA	N	5/12 (41.7%)	Poor
China(2022)	Ji et al. [33]	Y	Y	NR	Y	N	NA	NR	Y	Y	N	Y	NR	NA	N	6/12 (50%)	Fair
China (2022)	Li et al. [34]	Y	Y	Y	Y	N	NA	NR	N	Y	N	Y	NR	NA	Y	7/12 (58.3%)	Fair
China (2022)	Yang et al. [36]	N	Y	NR	NR	N	NA	Y	Y	Y	N	Y	NR	NA	NR	5/12 (41.7%)	Poor
China (2022)	Zhao et al. [37]	Y	Y	NR	NR	N	NA	N	N	N	Y	Y	NR	NA	Y	5/12 (41.7%)	Poor
China (2023)	Jiang et al. [38]	N	N	NR	Y	N	NA	NR	Y	N	N	Y	NR	NA	Y	4/12 (33.3%)	Poor

China (2023)	Zhang et al. [40]	Y	Y	NR	Y	N	NA	NR	Y	Y	N	Y	NR	NA	Y	7/12 (58.3%)	Fair
Netherlands (2023)	Fenneman et al. [42]	N	Y	NR	Y	N	NA	NR	Y	Y	N	N	NR	NA	N	4/12 (33.3%)	Poor

Quality of included studies was assessed using the National Institutes of Health (NIH) Quality Assessment tool for Observational Cohort and Cross-Sectional Studies(https://www.nhlbi.nih.gov/health-pro/guidelines/in-develop/cardiovascular-risk-reduction/tools/cohort). **1**. Was the research question or objective in this paper clearly stated? **2**. Was the study population clearly specified and defined? **3**. Was the participation rate of eligible persons at least 50%? **4**. Were all the subjects selected or recruited from the same or similar populations (including the same time period)? Were inclusion and exclusion criteria for being in the study prespecified and applied uniformly to all participants? **5**. Was a sample size justification, power description, or variance and effect estimates provided? **6**. For the analyses in this paper, were the exposure(s) of interest measured prior to the outcome(s) being measured? **7**. Was the timeframe sufficient so that one could reasonably expect to see an association between exposure and outcome if it existed? **8**. For exposures that can vary in amount or level, did the study examine different levels of the exposure as related to the outcome (e.g., categories of exposure, or exposure measured as continuous variable)? **9**. Were the exposure measures (independent variables) clearly defined, valid, reliable, and implemented consistently across all study participants? **10**. Was the exposure(s) assessed more than once over time? **11**. Were the outcome measures (dependent variables) clearly defined, valid, reliable, and implemented consistently across all study participants? **12**. Were the outcome assessors blinded to the exposure status of participants? **13**. Was loss to follow-up after baseline 20% or less? **14**. Were key potential confounding variables measured and adjusted statistically for their impact on the relationship between exposure(s) and outcome(s)?

Total Score: Number of yes; **NA,** not applicable; **NR,** not reported; **N,** no; **Y,** yes.

Quality Rating: Poor <50%, Fair 50-75%, Good ≥75%
