# Supplementary material for: Gut and Orbital Dysbiosis Associated with Graves’ Disease and Graves’ Orbitopathy: A Systematic Review
Source: J Clin Med. 2026 Jun 12;15(12):4586. doi: 10.3390/jcm15124586 (PMC13301356; doi:10.3390/jcm15124586)
Supplement: Supplementary file 1 [file jcm-15-04586-s001.zip › Table S5.pdf]

Table S5: Characteristics of Included Studies Investigating Dysbiosis in Graves' Disease and/or Graves' Ophthalmopathy (extended version)

Authors	Country	Study Design	Thyroid condition	Main objective	Thyroid parameters	Treatment features
**Ishaq et al. [18]**	Pakistan and China (2018)	Case-control	GD	Evaluate the diversity and similarity of intestinal microbiota qualitatively and quantitatively in GD as compared to their healthy counterparts	FT3, FT4, TSH, TRAb, Anti-TgAb, and anti-TPO	Untreated
**Shi et al. [19]**	China (2019a)	Case-control	GO	Investigate whether GO patients differ from healthy controls in the fecal microbiota	TT3, TT4, FT3, FT4, TSH, TRAb, Anti-TgAb, and anti-TPO	MMI
**Shi et al. [20]**	China (2019b)	Cross-sectional	GO	Explore the relationships between gut microbiota and GO-related traits	TT3, TT4, FT3, FT4, TSH, TRAb, Anti-TgAb, and anti-TPO	MMI
**Yang et al. [21]**	China (2019)	Case-control	GD	Explore the association of intestinal flora alteration with the development of GD among the Han population in southwest China	N/D	N/D
**Yan et al. [22]**	China (2020)	Case-control	GD	Investigate changes in intestinal flora that may occur in the setting of GD	FT3, FT4, TSH, TRAb, Anti-TgAb, and anti-TPO	Untreated
**Su et al. [23]**	China (2020)	Case-control	GD	Investigate the association and mechanism between intestinal flora and GD	FT3, FT4, TSH, TRAb, Anti-TgAb, and anti-TPO	Untreated
**Sun et al. [24]**	China (2020)	Prospective	GD	Observe changes in the gut microbiota structure caused by ATDs	FT3, FT4, TSH, TRAb, anti T anti-TPO	MMI PTU
**Cornejo-Pareja et al. [25]**	Spain (2020)	Case-control	GD + HT	Investigate the possible relationship between gut microbiota composition and the most frequent AITD	FT3, FT4, TSH, TRAb, anti-TPO	MMI

**Zhu et al. [26]**	China (2021)	Case-control	GD	Describe the intestinal microbial characteristics and microbial mutations of GD patients	FT3, FT4, TSH, TRAb, anti-TPO	Untreated
**Chang et al. [27]**	China (2021)	Case-control	GD	Characterize the composition of gut microbiota in GD patients	FT4, TSH, TRAb, anti-TPO	PTU or MMI or CBZ
**El-Zawawy et al. [28]**	Egypt (2021)	Case-control	GD + HT	Elucidate changes in gut microbiome in Egyptian patients with ATD	FT3, FT4, TSH, TRAb, anti TPOAb	10 untreated-GD<br>03 ATD
**Chen et al. [29]**	China (2021)	Prospective	GD	Investigate the correlation between human gut microbiota and clinical characteristics and thyroidal functional status of GD	N/D	MMI
**Huo et al. [30]**	China (2021)	Prospective	GD	Evaluate the curative effects of probiotics supplied with MI on thyroid function of patients with GD	FT3, FT4, TSH, TRAb	MMI: 20 mg/d<br>Black bean=100mg/d<br>Probiotic<br>Bifidobacterium longum: 2×10^7 CFU /d
**Jiang et al. [31]**	China (2021)	Case-control	GD	Examine the makeup and metabolic function of microbiota in GD patients	FT3, FT4, TSH, TRAb, Anti-TgAb, and anti-TPO	Untreated
**Shi et al. [32]**	China (2021)	Case-control	GD + GO	Identify specific intestinal bacteria of GD and GO, respectively	FT3, FT4, TSH, TRAb, Anti-TgAb, and anti-TPO	MMI-treated GD<br>Untreated GO
**Ji et al. [33]**	China (2022)	Case-control	GO	Investigate the diversity and composition of the ocular microbiota in patients with GO	N/D	ATD
**Li et al. [34]**	China (2022)	Case-control	GO	Investigate whether bacteria were present in the orbital adipose tissue of subjects with GO and if the amount and composition of these bacteria were correlated with the disease phenotypes.	N/D	N/D
**Han et al. [35]**	China (2022)	Prospective	GD	Explore the mechanism by which the combination of MMI and berberine may regulate the intestinal microbiota of patients with GD	FT3, FT4, TSH, and TRAb	MMI: 20 mg/d
**Yang et al. [36]**	China (2022)	Case-control	GD	Analyze the relationships between changes in the intestinal flora, thyroid function, and relevant thyroid antibodies in GD patients before and after MMI treatment	FT3, FT4, TSH, TRAb, Anti-TgAb, and anti-TPO	Patients from the treatment arm received 10-30mg/d MMI
**Zhao et al. [37]**	China	Case-control	GD + HT	Explore the role of gut microbiota in GD and HT	FT3, FT4, TSH,	Untreated

	(2022)				TRAb, Anti-TgAb, and anti-TPO	
**Jiang et al. [38]**	China (2023)	Case-control	GD + HT	Identify specific microbiota and metabolites that could distinguish Graves' disease patients, hypothyroidism patients, and controls	FT3, TT3, FT4, TT4, TSH, and TRAb	Untreated
**Deng et al. [39]**	China (2023)	Prospective	GD	Profile the gut microbiota of patients newly diagnosed with GD before and after treatment	FT3, FT4, TSH, TRAb, Anti-TgAb, and anti-TPO	MMI 20-30 mg/day
**Zhang et al. [40]**	China (2023)	Case-control	GO	Explore the changes of gut microbiota in GO patients of different severity grades	FT3, FT4, TSH, TRAb, Anti-TgAb, and anti-TPO	Untreated
**Biscarini et al. [41]**	UK, Italy, Belgium, and Germany (2023)	Prospective	GD + GO	Compare the fecal microbiota in GD patients, with GO of varying severity, and HC	FT3, FT4, TSH, TRAb	ATD Untreated GO
**Fenneman et al. [42]**	Netherlands (2023)	Case-control	GO	Evaluate the hypothesis stating that enhanced intestinal permeability may aggravate orbital inflammation	FT3, FT4, TSH, TRAb	All GO patients under ATD + LT4

Anti-TgAb: Anti-Thyroglobulin Antibodies; Anti-TPO: Anti-Thyroid Peroxidase Antibodies; ATD: Antithyroid Drugs; CBZ: Carbimazole; FT3: Free Triiodothyronine; FT4: Free Thyroxine; GD: Graves' Disease; GO: Graves' Ophthalmopathy; HC: Healthy Controls; LT4: Levothyroxine; MMI: Methimazole; N/D: Not Determined; PTU: Propylthiouracil; TSH: Thyroid-Stimulating Hormone; TRAb: Thyroid Receptor Antibodies.
