# Supplementary material for: Gut and Orbital Dysbiosis Associated with Graves’ Disease and Graves’ Orbitopathy: A Systematic Review
Source: J Clin Med. 2026 Jun 12;15(12):4586. doi: 10.3390/jcm15124586 (PMC13301356; doi:10.3390/jcm15124586)
Supplement: Supplementary file 1 [file jcm-15-04586-s001.zip › Table S6.pdf]

Table S6: Gut Dysbiosis in Patients with Graves' Disease: Alpha and Beta Diversities, and Taxonomic Composition (extended version)

1st Autor	Sample Size	Age (yrs)	F (%)	Site	Microbiota Acquisition	Alpha Diversity Indices	Alpha Diversity	Beta Diversity Analysis	Beta Diversity	Taxonomic composition		Additional Findings
										Phylum Level	Genus Level	
**Ishaq et al. (2018) [18]**	27 GD<br>11 HC	35-50<br>-	63%<br>64%	Fecal	Amplification of the 16S rRNA gene (V3-V4 variable region)	Simpson<br>Shannon<br>ACE<br>Chao1	The α diversity was significantly reduced in GD group compared with HC group.	Dice similarity coefficient<br>UPGMA (weighted UniFrac distance)	Gut microbiota present a separate structure from HC.	In GD group the F/B ratio was lower than in HC group.<br>There was more prevalence of Bacteroidetes, Actinobacteria, and Proteobacteria and less Firmicutes in the GD patients than in the healthy group.	Compared to HC, the genera Prevotella_9 and Haemophilus were increased in GD patients, while Bacteroides, Ruminococcus, Dialister, and Alistipes were reduced.	
**Yang et al. (2019) [21]**	15 GD<br>15 HC	46-55<br>-	-<br>-	Fecal	Amplification of the 16S rRNA gene	Chao1<br>ACE<br>Shannon<br>Simpson	The diversity indices in GD group were lower than those for the control group	PLS-DA	The GD samples were separated from those of HC	The F/B ratio was significantly higher in GD cases when compared with HC.<br>The GD group showed significantly higher abundances of Firmicutes, Proteobacteria and Actinobacillus.	The abundances of the genera Oribacterium, Mogibacterium, Lactobacillus, Aggregatibacter and Mogibacterium were significantly higher in the GD group than in the control group.<br>Prevotella was more increased in the GD group than in HC group, although the difference was not significant.	
**Yan et al. (2020) [22]**	39 GD<br>07 HC	37<br>33	72%<br>65%	Fecal	Amplification of the 16S rRNA gene	Observed Species<br>Chao1<br>Shannon	There was lower richness and evenness in the intestinal microbiota of GD	PCA, PCoA, NMDS	The intestinal flora clustering of the two groups showed distinct distribution from each other, without	N/D	Bacilli, Lactobacillales, Prevotella, Megamonas and Veillonella strains were increased, whereas Ruminococcus,	

						Simpson	patients compared to HC.		displaying any intersections.		Rikenellaceae and Alistipes strains were decreased among patients with GD.	
**Su et al. (2020) [23]**	63 GD<br>58 HC	44<br>42	56%<br>60%	Fecal	Amplification of the 16S rRNA gene (V1-V2 variable region)	ACE<br>Chao1<br>Shannon<br>Simpson<br>Pielou index	There was a significantly decreased α diversity of the intestinal flora in GD patients than in HC.	PCoA<br>NMDS (Bray-Curtis distance)	The microbiota composition differed significantly between the two groups	The F/B ratio decreased significantly in GD patients.<br>The abundance of other bacterial phyla, including Proteobacteria, Saccharibacteria, and Verrucomicrobia in GD patients, were also markedly changed compared with those in the HC.	The random forest analysis also showed that 3 intestinal bacteria (Bacteroides, Alistipes, Prevotella) could distinguish GD patients from healthy individuals with 85% accuracy.<br>Yersinia enterocolitica was significantly higher in GD patients with diarrhea than in GD patients without diarrhea and HC.	The Pielou and Simpson indexes were significantly negatively correlated with the serum levels of TRAb, FT3, and FT4, indicating a significant association with the intensity of the disease.
**Sun et al. (2020) [24]**	40 GD<br>50 HC	37<br>38	N/D<br>N/D	Fecal	Amplification of the 16S rRNA gene (V3-V4 variable region)	Observed OUT<br>ACE<br>Shannon	The richness increased in GD patients compared with HC. It was higher in GD after the ATD therapy<br>The evenness increased in GD after treatment. However in both instances, it was still lower than in HC.	PCoA (Bray-Curtis distance)	GD group deviated from the HC group.<br>After treatment, The GD group showed a greater deviation from the HC group and partially deviated from its previous composition before treatment.	The F/B ratio was significantly higher in GD patients before ATD therapy than after the treatment. Treated GD had a higher F/B ratio than HC.	The abundance of Faecalibacterium and Clostridium_sensu_stricto_1 decreased after ATD therapy, while Eubacterium_rectale, Romboutsia and Dorea showed a higher abundance in treated patients.	ATD altered gut microbiota structure
**Cornejo-Pareja et al. (2020) [25]**	09 GD<br>11 HC	46<br>49	78%<br>64%	Fecal	Amplification of the 16S rRNA gene (V2-V3 variable region)	Observed OUT<br>Shannon<br>Pielou index	The bacterial richness was comparable between GD and HC groups. The evenness decreased in GD patients compared with HC.	PCoA (unweighted and weighted UniFrac distances)	Beta diversity analysis showed that gut microbiota from both groups was different.	No significant difference in the F/B ratio was observed between the two groups.	Fusobacterium was significantly higher in GD patients compared with HC. Faecalibacterium was significantly lower in GD patients compared to HC.<br>The Prevotella genus seemed to be characteristic of the GD group	TRAb level was positively correlated with Lactobacillus and Pasteurellaceae but negatively correlated with Faecalibacterium.

**Zhu et al. (2021) [26]**	100 GD<br>36 Mild GD<br>64 Severe GD<br>62 HC	44<br><br><br>40	60%<br><br><br>47%	Fecal	Shotgun metagenomic sequencing	Shannon<br>Simpson	There was a decrease in microbial α diversity in patients with severe GD.	PCoA (Aitchison distance, Bray–Curtis dissimilarity)	Intestinal microbiota in the HC and mild GD groups were similar but obviously separated from the patients in the severe GD group.	N/D	Coprobacillus, streptococcus, and Rothia are enriched in all GD patients.<br>Faecalibacterium prausnitzii, Butyricimonas faecalis, Bifidobacterium adolescentis and Akkermansia muciniphila decreased in the severe GD group.	
**Chang et al. (2021) [27]**	55 GD<br>48 HC	45<br>43	67%<br>63%	Fecal	Amplification of the 16S rRNA gene (V3-V4 variable region)	ACE<br>Chao1<br>Shannon<br>Simpson	The microbial richness and evenness of GD group were similar to that of the HC.	PCoA<br>PLS-DA	The overall community structure was distinctive between the two sample groups.	The F/B ratio was significantly lower in GD group than in the controls.<br>The relative abundance of the phyla Bacteroidetes and Actinobacteria was increased, while Firmicutes decreased in the GD group compared to the HC.	The abundances of Bacteroides and Prevotella_9 were significantly higher, while Faecalibacterium and Lachnospiraceae_NK4A136_group were slightly lower in the GD group compared to the HC.	
**El-Zawawy et al. (2021) [28]**	13 GD<br>30 HC	38<br>30	69%<br>57%	Fecal	Amplification of the 16S rRNA gene	Shannon	No significant difference in α diversity was observed between the two groups.	Bray-Curtis similarity index	The similarity in gut microbiota between GD and HC groups was 68%.	The F/B ratio was significantly lower in GD cases when compared with HC .	Patients with GD showed a significant increase in Prevotella compared to HC.	TRAb in GD patients showed a significant positive correlation with Bacteroidetes.
**Chen et al. (2021) [29]**	15 GD<br>14 HC	27<br>29	-<br>-	Fecal	Amplification of the 16S rRNA gene (V3-V4 variable region)	Observed OUT<br>Chao1<br>Shannon<br>Simpson	The abundance and diversity of gut microbiota were significantly reduced in GD patients, while increased apparently after treatment along with the improvement of microflora.	N/D	The composition and structure of gut microbiota between GD patients and healthy volunteers were significantly different.	The F/B ratio was lower in untreated GD patients compared to HC.<br>The relative abundances of Proteobacteria and Synergistetes in GD patients were significantly reduced compared with the HC group.<br>The Proteobacteria significantly increased in treated GD patients compared with the same group before ATD.	The relative abundance of Lactobacillus, Veillonella and Streptococcus increased significantly in GD. After the improvement of thyroid function, a significant reduction at the genus level as observed in Blautia, Corynebacter, Ruminococcus and Streptococcus, while Phascolarctobacterium increased significantly.	The correlation between the TRAb level and the relative abundance of Lactobacillus and Ruminococcus was positive.<br>Synergistetes and Phascolarctobacterium showed a negative correlation with

												TRAb.
**Huo et al. (2021) [30]**	26 GD:<br>08 treated with MMI<br>09 treated with MMI+ black-bean<br>09 treated with MMI+ probiotic Bifidobacterium longum	N/D	N/D	Fecal	Shotgun metagenomic sequencing	Shannon<br>Simpson	A decline was observed in the microbial diversity between baseline samples and those treated for 6 months with MMI.	PCoA (Bray-Curtis distance)	MMI intake led to alterations in intestinal microbiota in patients with GD from ecological and evolutionary perspectives<br>Black-bean adjuvant MMI intake maintained intestinal microbiome homeostasis in patients with GD during the 6-month treatment.	This aspect was not studied	In patients treated with MMI, Faecalibacterium prausnitzii, Ligilactobacillus salivarius, Lactococcus lactis, and some species of the genera Porphyromonas a nd Prevotella significantly decreased.	Probiotic Bifidobacterium longum adjuvant methimazole treatment improved thyroid function and significantly reduced the TRAb concentration of patients with GD.
**Jiang et al. (2021) [31]**	45 GD<br>59 HC	37<br>42	73%<br>63%	Fecal	Amplification of the 16S rRNA gene (V3-V4 variable region)	SOBs<br>ACE<br>Chao1<br>Shannon<br>Simpson	GD patients had reduced diversity and abundances of certain microbiota compared to control subjects.	PLS-DA (unweighted UniFrac distance)	The microbial composition of GD patients was significantly different than that of controls	The F/B ratio was lower in untreated GD patients compared to HC.	GD patients had greater numbers of Faecalibacterium, Bacteroides, Prevotella_9, and Bifidobacterium and lower numbers of Blautia, Subdoligranulum, [Eubacterium]_rectale_group.	Bacteroides, Blautia, [Eubacterium]_hallii_group, Anaerostipes, Lactobacillus, Dorea could serve as diagnostic biomarkers according to random forest analysis.
**Han et al. (2022) [35]**	08 GD treated with MMI	-	-	Fecal	Shotgun metagenomic sequencing	Shannon<br>Simpson	The α diversity of the gut microbiota of the patients showed a	PCoA (Bray-Curtis distances)	There was a significant change in the structure of patients' gut microbiota after 6	N/D	MMI alone failed to modulate the gut microbiota of the patients. However, the combination	

	10 GD treated with MMI+ Berberine						tendency to decrease and then increase after MMI treatment supplemented with berberine		months compared to baseline.<br>The addition of berberine reshaped the structure of the patients' gut microbiota in contrast to MMI alone.		of berberine with MMI significantly shifted the microbiota structure of the patients, increasing the abundance of the beneficial bacteria Lactococcus lactis while decreasing the abundance of the pathogenic bacteria Enterobacter hormaechei and Chryseobacterium indologenes. In addition, it significantly reduced the abundance of Prevotella spp.	
**Yang et al. (2022) [36]**	18 Untreated GD<br>10 Treated GD<br>11 HC	37<br>30<br>30	61%<br>60%<br>55%	Fecal	Amplification of the 16S rRNA gene (V3-V4 variable region)	Chao1<br>ACE<br>Shannon<br>Simpson	The biological diversity of the intestinal flora was reduced in patients with GD compared to those in the HC group<br>It was higher in the untreated GD group than in the treated GD group.	NMDS (Unweighted UniFrac distance)	There were certain overlaps among the three groups.	The F/B ratio was higher in HC compared to patients with GD.<br>Relative to the HC group, Actinobacteria, Cyanobacteria, and TM7 were significantly increased in the untreated GD group, while Firmicutes and [Thermi] were decreased.<br>Relative to the HC group, Proteobacteria and TM7 were significantly increased in the treated GD group, while [Thermi] was decreased.<br>The relative abundance of Actinobacteria in the untreated GD group was higher than that in the treated GD group .	In the untreated GD group, the Collinsella abundance was significantly higher than that in the HC group and the treated GD group.<br>In untreated GD patients, the Bifidobacterium abundance was significantly higher and the Dialister and Roseburia abundances were significantly lower than those in the HC group.<br>The Prevotella abundance in the treated GD group was lower than those in the HC group and untreated GD group.	
**Zhao et al. (2022) [37]**	27 GD<br>16 HC	49<br>49	70%<br>56%	Fecal	Amplification of the 16S rRNA gene (V3-V4 variable region)	Observed OUT<br>Shannon<br>Simpson<br>SOBs	The diversity of gut microbiota in the HC group tended to be lower than in the GD group	PLS-DA (Jaccard similarity)	The overall structure of the gut microbiota was significantly different between the GD group and HC group,	Proteobacteria and Firmicutes increased in GD patient samples. The proportions of Cyanobacteria in the GD samples were higher than those in the HC samples	Prevotella_9, Ruminococcus_2, and Lachnospiraceae_NK4A136_group were higher in GD patient samples compared to HC samples.	The authors suggested that Bacillus, Blautia, and Ornithinimicrobium could be used as potential markers to

												distinguish GD from HC.
**Jiang et al. (2023) [38]**	39 GD<br>48 HC	40<br>45	69%<br>63%	Fecal	Amplification of the 16S rRNA gene (V3-V4 variable region)	SOBs<br>Chao1<br>Shannon	The α diversity was significantly lower in GD group than in controls.	PLS-DA	Gut microbiota tends to cluster by group, reflecting the significant distinction in microbial composition between GD and HC groups.	The F/B ratio was significantly lower in GD group than in the controls.	The proportions of Bacteroides and Lactobacillus were significantly increased in GD patients than in the controls, while the proportions of Blautia, [Eubacterium]_hallii_group, and Collinsella were significantly decreased.	
**Deng et al. (2023) [39]**	65 GD<br>33 HC	30<br>27	72%<br>70%	Fecal	Amplification of the 16S rRNA gene (V3-V4 variable region)	Observed ASV<br>Faith's phylogenetic diversity<br>Shannon<br>Pielou index	The α diversity analysis showed significantly reduced richness and evenness in the GD group compared to the HC group.	PLS-DA<br>PCOA (Bray-Curtis distance)	The gut microbiota composition of patients with GD was significantly different from that of healthy controls.	No significant difference in the F/B ratio was observed between the two groups	Streptococcus, Veillonella, and Erysipelatoclostridium were significantly enriched in GD patients. However, the abundances of Roseburia, Romboutsia, Lachnospira, and Eubacterium ventriosum were significantly lower in the GD group than in the Control group.	The intestinal flora of patients with GD after treatment had been gradually reconstructed and recovered.

ACE: Abundance-based Coverage Estimator; ASV: Amplicon Sequence Variant; ATD: Antithyroid Drug; BMI: Body Mass Index; F: Female; FT3: Free Triiodothyronine; FT4: Free Thyroxine; GD: Graves' Disease; HC: Healthy Controls; MMI: Methimazole; NMDS: Non-metric Multidimensional Scaling; N/D: Not Determined; OTU: Operational Taxonomic Unit; PCA: Principal Component Analysis; PCoA: Principal Coordinates Analysis; PLS-DA: Partial Least Squares Discriminant Analysis; SOBs: Species Observed; TRAb: Thyrotropin Receptor Antibody; UPGMA: Unweighted Pair Group Method with Arithmetic Mean; yrs: Years.
