# Supplementary material for: Gut and Orbital Dysbiosis Associated with Graves’ Disease and Graves’ Orbitopathy: A Systematic Review
Source: J Clin Med. 2026 Jun 12;15(12):4586. doi: 10.3390/jcm15124586 (PMC13301356; doi:10.3390/jcm15124586)
Supplement: Supplementary file 1 [file jcm-15-04586-s001.zip › Table S7.pdf]

Table S7: Gut and Orbital Dysbiosis in Patients with Graves' Orbitopathy: Alpha and Beta Diversities, and Taxonomic Composition (extended version)

Authors	Sample Size	Age (yrs)	F (%)	Site	Microbiota Acquisition	Alpha Diversity Indices	Alpha Diversity	Beta Diversity Analysis	Beta Diversity	Taxonomic composition		Additional Findings
										Phylum Level	Genus Level	
**Shi et al. (2019a) [19]**	33 GO<br>32 HC	46<br>43	52%<br>50%	Fecal	Amplification of the 16S rRNA gene	Simpson<br>Shannon<br>Chao1<br>ACE	The α diversity was significantly reduced in patients with GO as compared to HC.	PCoA (weighted UniFrac distance)	The fecal microbiota of the patients with GO and HC were significantly separated despite the inter-individual variation.	The proportion of Bacteroidetes increased significantly in patients with GO as compared to HC while the proportion of Firmicutes decreased markedly in patients with GO as compared to HC.	The proportion of Prevotellaceae was significantly increased while that of Blautia, Fusicatenibacter, Butyricicoccus, Anaerostipes, and Collinsella was markedly decreased in patients with GO as compared to the controls.	The proportion of Succinivibrionaceae was positively correlated to TRAb.
**Shi et al. (2019b) [20]**	31 GO	45	52%	Fecal	Amplification of the 16S rRNA gene	N/D	N/D	Not applicable	Not applicable	Firmicutes and Bacteroidetes, as the most predominant phyla in GO patients. The proportion of Bacteroidetes in the top ten OTUs is very high.	N/D	The genera, s_Prevotella_copri and f_Prevotellaceae, showed a significant positive correlation withTRAb.

**Shi et al. (2021) [32]**	30 GD<br>33 GO<br>32 HC	45<br>46<br>43	67%<br>48%<br>50%	Fecal	Amplification of the 16S rRNA gene	Observed OUT<br>Chao1<br>ACE<br>SOBs<br>Shannon<br>Simpson	The Shannon diversities were reduced in patients with GD and GO as compared to HC. However, no significant difference was detected in Chao1 and ACE indices.	PCoA (weighted UniFrac distance)	The intestinal bacteria of the patients with GD and GO were significantly separated from HC despite the inter-individual variation	The F/B ratio was higher in HC than in GD and GO patients.<br>The proportion of Bacteroidetes increased significantly in patients with GO as compared to the HC, while the proportion of Firmicutes decreased significantly in patients with GO as compared to HC.	The proportion of unidentifed_Prevotellaceae was significantly increased while that of Blautia, Fusicatenibacter, Butyricicoccus, Anaerostipes, and Collinsella was markedly decreased in patients with GO as compared to the controls.<br>The proportion of Subdoligranulum and Bilophila was increased while that of Blautia, Anaerostipes, Dorea, Butyricicoccus, Romboutsia, Fusicatenibacter, unidentifed_Lachnospiraceae, unidentifed_Clostridiales, Collineslla, Intestinibacter, and Phascolarctobacterium was decreased in the GO group as compared to the GD group.	
**Ji et al. (2022) [33]**	67 GO<br>22 HC	44<br>63	58%<br>68%	Ocular surface	Amplification of the 16S rRNA gene (V3-V4 variable region)	Shannon index	There was no significant difference in α diversity between the GO and control groups.	PCoA (Bray-Curtis dissimilarity matrix)	There was no significant aggregation difference between the two groups	The composition of dominant bacteria on the ocular surface of the two groups was the same, in the order of Proteobacteria, Firmicutes, Actinobacteria, and Bacteroidetes.<br>Compared with the control group, Proteobacteria abundance in the GO group was significantly higher. The abundance of Firmicutes and Actinobacteria was lower than that in the control group.<br>Acidobacteriota and Verrucomicrobiota were more prevalent in the ocular surface of patients with GO	Bacillus and Brevundimonas increased significantly in the GO group. Corynebacterium had a significantly decreased relative abundance.	Paracoccus, Haemophilus, Lactobacillus, and Bifidobacterium were positively correlated with the severity of clinical manifestations or disease activity.
**Li et al. (2022) [34]**	27 GO<br>27 HC	48<br>25	67%<br>37%	Orbital adipose tissue	Amplification of the 16S rRNA gene	Observed OTU	GO orbital fat microbiota diversity was significantly lower than in controls	PCA	There were significant differences between GO patients and controls	Bacteroidetes were enriched in GO and Firmicutes in controls	The proportions of Pseudomonas, Comamonas, Brevundimonas, Aeromonas, Flavobacterium and Janthinobacterium were significantly higher in GO than those in control.	

**Zhang et al. (2023) [40]**	62 GO:<br>20 mild<br>25 moderate<br>17 severe<br>18HC	 38<br>40<br>55<br>34	 65%<br>68%<br>30%<br>83%	Fecal	Amplification of the 16S rRNA gene (V3-V4 variable region)	Observed OUT<br>Chao1<br>ACE<br>SOBs<br>Shannon	No significant difference in gut microbiota α diversity between the groups.	PCA and NMDS	The gut microbial community between the control and GO groups differed significantly.	The F/B ratio among the four groups showcased no difference	Compared with the mild GO group, the abundance of Faecalibacterium_prausnitzii was increased in the moderate to severe group, and Klebsiella_pneumoniae abundance was increased in the sight-threatening group.	Klebsiella_pneumoniae was a potential GO-related pathogen, which may regulate the metabolic pathways to affect GO progression.
**Biscarini et al. (2023) [41]**	59 GD<br>46 GO<br>41 HC	46<br>47<br>46	90%<br>87%<br>78%	Fecal	Amplification of the 16S rRNA gene (V1-V2 variable region)	Observed OUT<br>Chao1<br>Shannon<br>Equitability	N/D	NMDS (Bray-Curtis dissimilarity matrix)	N/D	The F/B ratio was significantly increased in GD versus HC and in all cases (GD and GO).<br>Actinobacteria phylum significantly increased in GD and GO compared to controls and were significantly more abundant in GO than in GD.<br>Bacteroidetes significantly decreased in GD and GO compared to HC.	Bacteroides spp were significantly decreased in GD and GO compared to HC, while Fusicatenibacter spp was enriched in GD and GO compared to HC.<br>Reduced Bacteroides spp , increased Bifidobacterium spp, and increased Fusicatenibacter spp were significantly associated with mild GO, but not with moderate-severe GO.<br>Roseburia spp was enriched in moderate-severe GO compared to HC, GD, and mild GO.	Bacteroides spp represented one of the top bacterial biomarkers when predicting GO severity.<br>All genera uniquely associated with TRAb were Firmicutes of the Clostridiales family.
**Fenneman et al. (2023) [42]**	57 GO<br>42 non-operated<br>15 operated<br>15 HC (operated)	46<br><br><br>42	67%<br><br><br>54%	Fecal<br>Orbital adipose tissue samples (for operated GO)	Amplification of the 16S rRNA gene (V3 variable region)	N/D	N/D	N/D	N/D	N/D	The relative abundance of two Gram-negative species, Bacteroides spp. and Dialister spp., was positively correlated with the concentration of serum lipopolysaccharide-binding protein, linking the gut to local orbital inflammation.	Lactobacillus abundance in stool samples was shown to be associated with the severity of GO and specifically with orbital adipogenesis.

ACE: Abundance-based Coverage Estimator; ASV: Amplicon Sequence Variant; F: Female; GO: Graves' Orbitopathy; HC: Healthy Controls; N/D: Not Determined; NMDS: Non-metric Multidimensional Scaling; OTU: Operational Taxonomic Unit; PCA: Principal Component Analysis; PCoA: Principal Coordinates Analysis; PLS-DA: Partial Least Squares Discriminant Analysis; SOBs: Species Observed; TRAb: Thyrotropin Receptor Antibody; yrs: Years.
